# Beyond the COVID‐19 pandemic: increasing the uptake of influenza vaccination by health and aged care workers

**DOI:** 10.5694/mja2.51541

**Published:** 2022-05-14

**Authors:** Lyn‐li Lim, Noleen J Bennett, Sheena G Sullivan, Ann L Bull, Leon J Worth

**Affiliations:** ^1^ Victorian Healthcare Associated Infection Surveillance System Coordinating Centre the Peter Doherty Institute for Infection and Immunity Melbourne VIC; ^2^ The University of Melbourne Melbourne VIC; ^3^ National Centre for Antimicrobial Stewardship the Peter Doherty Institute for Infection and Immunity Melbourne VIC; ^4^ WHO Collaborating Centre for Reference and Research on Influenza Melbourne VIC

**Keywords:** Vaccination, Influenza, Health policy

In Victoria, public acute health care facilities (hospitals and their health services) and residential aged care services (RACS) are responsible for staff vaccination programs. The influenza vaccination target for health care facility staff was set at 75% in 2014, and incrementally raised to 92% in 2021; influenza vaccination of RACS staff was made mandatory throughout Australia from May 2020.^1^ In this article, we report the vaccination rates (by calendar year) during the coronavirus disease 2019 (COVID‐19) pandemic (2020 and 2021), and compare them with rates for 2018 and 2019.

Information about health care staff vaccination is submitted by all health care facilities and RACS to the Victorian Healthcare Associated Infection Surveillance System Coordinating Centre (VICNISS; https://www.vicniss.org.au). As we analysed aggregate non‐identifiable data for quality improvement purposes, consistent with quality assurance activities defined according to National Health and Medical Research Council recommendations,^2^ formal ethics approval was not required for our study.

The influenza vaccination rate in acute health care facilities rose from 83.2% in 2018 to 93.0% in 2020, but fell to 77.4% in 2021; in RACS, it rose from 86.9% in 2018 to 98.9% in 2020, but fell to 88.1% in 2021. The proportion of staff who declined vaccination was larger in 2021 than in 2020, but smaller than in 2018 or 2019. The proportion of acute health care facility staff with undeclared vaccination status increased from 4.1% in 2020 to 18.3% in 2021 (Box).

Box 1 **Influenza vaccination uptake by staff members (clinical and non‐clinical) in public acute health care facilities and residential aged care services, Victoria, 2018–2021**

**Table:** 

	Pre‐COVID‐19 pandemic period		COVID‐19 pandemic period	
Characteristic	2018	2019	2020	2021
**Hospitals and their health services**				
Number of facilities	103	105	112	101
Number of staff members	120 483	125 167	135 211	141 181
Vaccinated	100 239 (83.2%)	109 750 (87.7%)	125 796 (93.0%)	109 251 (77.4%)
Declined vaccination	8219 (6.8%)	7556 (6.0%)	3904 (2.9%)	5615 (4.0%)
Vaccination medically contraindicated	0	0	0	545 (0.4%)
Vaccination status unknown/undeclared	0	0	5511 (4.1%)	25 770 (18.3%)
Uncategorised^†^	12 025 (10.0%)	7861 (6.3%)	0	0
**Residential aged care services**				
Number of facilities	177	175	177	174
Number of staff members	12 536	13 844	12 270	10 400
Vaccinated	10 894 (86.9%)	12 181 (88.0%)	12 136 (98.9%)	9163 (88.1%)
Declined vaccination	948 (7.6%)	1179 (8.5%)	94 (0.8%)	292 (2.8%)
Vaccination medically contraindicated	NA	NA	NA	38 (0.4%)
Vaccination status unknown/undeclared	NA	NA	40 (0.3%)	907 (8.7%)
Uncategorised^†^	694 (5.5%)	484 (3.5%)	0	0

NA = not available (data not collected).

* Acute care hospitals and their health service.

† That is: vaccination status was incomplete in the submitted data.

It is likely that influenza vaccination uptake by health and aged care workers declined in Victoria during 2021 because the focus on COVID‐19 risk mitigation activities, including the COVID‐19 vaccination program, affected staff influenza vaccination program activities, including workplace and after hours vaccination, and influenza vaccination promotion and reminders. Mobile carts may have been prohibited by COVID‐19‐related restrictions. Moreover, the low community prevalence of influenza during 2020 and 2021 may have led to complacency about vaccination.

The higher health care facility rate for 2020 than for 2018 and 2019 may have reflected campaigns promoting influenza vaccination to avoid a dual epidemic of COVID‐19 and influenza, whereas the larger rise in RACS was the result of the new vaccination mandate.

Although mandatory vaccination policies can achieve high, sustained rates,^3^ it reduces the autonomy and empowerment of staff members; they may feel pressured, and contend that individual choice should be respected.^4^ Mandatory policies should take basic ethical principles into consideration, including effectiveness (vaccine and program), beneficence (that this option is better than other options), autonomy (minimum infringement of personal choice), justice (protection of people in vulnerable groups), and transparency (public trust in the vaccination program).^5^

Staff members who refuse mandatory vaccination can face restrictions (eg, wearing masks) or redeployment. Enforcing mandatory policies may consequently adversely affect staffing capacity, recruitment, and morale.^6^ As a very high vaccination uptake has been achieved in Australian health care facilities without mandates, the potential negative consequences of compulsion require consideration.

If influenza activity increases in Australia during the 2022 winter, as is expected, it could significantly affect staff and result in health care‐associated influenza infections. It is therefore important that planning and support is provided to achieve very high influenza vaccination uptake by health care facility and RACS staff.

## Open access

Open access publishing facilitated by The University of Melbourne, as part of the Wiley ‐ The University of Melbourne agreement via the Council of Australian University Librarians.

## Competing interests

No relevant disclosures.

## References

[1] Minister for Senior Australians and Aged Care Services (Australia) . Aged care workers must get flu vaccination [media release]. 3 Apr 2020. https://www.health.gov.au/ministers/senator‐the‐hon‐richard‐colbeck/media/aged‐care‐workers‐must‐get‐flu‐vaccination (viewed May 2022).

[2] National Health and Medical Research Council . Ethical considerations in quality assurance and evaluation activities. Mar 2014. https://www.nhmrc.gov.au/about‐us/resources/ethical‐considerations‐quality‐assurance‐and‐evaluation‐activities (viewed Apr 2022).

[3] Schumacher S , Salmanton‐García J , Cornely OA , Mellinghoff SC . Increasing influenza vaccination coverage in healthcare workers: a review on campaign strategies and their effect. Infection 2021; 49: 387‐399. 33284427 10.1007/s15010-020-01555-9 PMC7720031

[4] National Institute for Health and Care Excellence . Flu vaccination: increasing uptake NICE Guideline (NG 103). Aug 2018. https://www.nice.org.uk/guidance/ng103 (viewed Apr 2022).

[5] Steckel CM . Mandatory influenza immunization for health care workers: an ethical discussion. AAOHN J 2007; 55: 34‐39 17260679 10.1177/216507990705500105

[6] National Institute for Health and Care Excellence . Flu vaccination: increasing uptake. Evidence reviews for RQ4&5 increasing uptake in health and social care workers [draft for consultation]. June 2017. https://www.nice.org.uk/guidance/gid‐phg96/documents/evidence‐review‐4 (viewed Apr 2022).

